# Hormonal Contraceptive Treatment May Reduce the Risk of Fibromyalgia in Women with Dysmenorrhea: A Cohort Study

**DOI:** 10.3390/jpm10040280

**Published:** 2020-12-14

**Authors:** Cheng-Hao Tu, Cheng-Li Lin, Su-Tso Yang, Wei-Chih Shen, Yi-Hung Chen

**Affiliations:** 1 Graduate Institute of Acupuncture Science, China Medical University, Taichung 404333, Taiwan; 2 Management Office for Health Data, China Medical University Hospital, Taichung 404332, Taiwan; orangechengli@gmail.com; 3 Department of Medical Imaging, China Medical University Hospital, Taichung 404332, Taiwan; yangst@mail.cmu.edu.tw; 4 School of Chinese Medicine, China Medical University, Taichung 404333, Taiwan; 5 Center of Augmented Intelligence in Healthcare, China Medical University Hospital, Taichung 404332, Taiwan; wcshen@gmail.com; 6 Department of Computer Science and Information Engineering, Asia University, Taichung 413305, Taiwan; 7 Traditional Chinese Medicine Research Center, China Medical University, Taichung 404333, Taiwan; 8 Department of Photonics and Communication Engineering, Asia University, Taichung 41354, Taiwan

**Keywords:** dysmenorrhea, fibromyalgia, risk factor, hormonal contraceptives

## Abstract

Dysmenorrhea is the most common gynecological disorder for women in the reproductive age. Study has indicated that dysmenorrhea might be a general risk factor of chronic pelvic pain and even chronic non-pelvic pain, such as fibromyalgia. We used the Longitudinal Health Insurance Database 2000 from the Taiwan National Health Research Institutes Database to investigate whether women with dysmenorrhea have a higher risk of fibromyalgia and whether treatment of dysmenorrhea reduced the risk of fibromyalgia. The dysmenorrhea cohort was matched with a non-dysmenorrhea cohort at a 1:1 ratio based on gender, age, and the year of entry study by frequency matching. Multivariable Cox proportional hazard regression models were used to assess the risk of fibromyalgia, with controlling for potential confounding variables such as age, comorbidities, and medication use. After controlling confounding variables, results revealed that women with dysmenorrhea have a significantly higher risk of fibromyalgia than women without dysmenorrhea. However, only treatment of dysmenorrhea with hormonal contraceptives reduce the risk of fibromyalgia. These results indicated that dysmenorrhea may be a risk factor of fibromyalgia, whereas personalized medicine for treatment of dysmenorrhea may be the key to reduce the risk of fibromyalgia. Future studies are needed to identify the causes and prevention strategies in detail.

## 1. Introduction

Dysmenorrhea is the most common gynecological disorder for women in the reproductive age. Epidemiological studies have shown that 16% to 91% of female have experienced dysmenorrhea, and 2% to 29% of female have had severe dysmenorrhea [1]. Dysmenorrhea patients suffered from cramping pain emanating from the lower abdomen when the menstruation onset [2], and often accompany with other symptoms such as headaches, depression, anxiety, and insomnia [1,3,4]. Previous study reported that the severity of dysmenorrhea negatively affected the live quality of patients [5]. Clinically, dysmenorrhea can be characterized as primary (menstrual pain without pelvic abnormality) or secondary (menstrual pain with pelvic abnormality, e.g., endometriosis) [6]. The first-line pharmacological treatment for primary dysmenorrhea is nonsteroidal anti-inflammatory drugs (NSAID). If the NSAID treatment is ineffective to management dysmenorrhea, treatment with hormonal contraceptives or antidepressants can be considered [6,7]. For secondary dysmenorrhea with endometriosis, the first-line pharmacological treatment is oral contraceptive pill [6]. However, it has been reported that both treatment with NSAID and treatment with oral contraceptive pill have less effectiveness for severe dysmenorrhea than for mild dysmenorrhea [8]. Moreover, study has indicated that dysmenorrhea might be a general risk factor of chronic pelvic pain and even chronic non-pelvic pain [9]. Thus, dysmenorrhea greatly impacts the women health and should be more considered in pain management.

Fibromyalgia is an idiopathic chronic pain condition with a higher prevalence rate in women than in men. In the United States, it has been estimated that 2.38% women have fibromyalgia, whereas only 1.06% men have fibromyalgia [10]. Fibromyalgia patients have generalized body pain and other somatic and psychological symptoms [11], which greatly impact their quality of life [12]. The risk factors of fibromyalgia may include sex, age, smoking, body mass index, alcohol consumption, and pre-existing medical disorders [13]. Among these risk factors, the strongest risk factors are sleep disorder, headaches and other pains, anxiety/depression, and illness behavior. Clinically, treatment of fibromyalgia is multidisciplinary. The initial treatment should be non-pharmacological therapies, then followed with pharmacotherapy if the patient has no response to the initial treatment [14]. The pharmacotherapy of fibromyalgia was antidepressants and anticonvulsants, whereas NSAID was ineffective to management fibromyalgia [14,15]. Previous studies have reported that a higher prevalence rate of dysmenorrhea among women with fibromyalgia than women with arthritis and healthy controls indicated a high comorbidity between dysmenorrhea and fibromyalgia [16,17]. However, the underling mechanisms which make dysmenorrhea highly comorbid with fibromyalgia still unclear.

An attractive theory is that prior pain experience may increase the pain vulnerability in individual, which may increase the risk of developing chronic pain condition [18]. Combined with predisposing factors in the brain, the acute nociceptive signals from the peripheral will be manipulated by the mesolimbic learning mechanisms and then influence information exchanges between the prefrontal cortex and limbic system that decides pain chronification [19]. The successful adaptation to nociceptive inputs may not lead to the chronification of pain, while maladaptation to nociceptive inputs may lead to the chronification of pain. Indeed, previous brain imaging studies have reported that dysmenorrhea patients who developed with chronic pelvic pain have maladaptive changes in central pain system, whereas dysmenorrhea patients who have no chronic pelvic pain revealed adaptive changes in central pain system [20]. The structural alterations associated with menstrual pain in the brain may be the results of cumulative effect from repetitive rapid neuroplasticity [21]. Hence, with a greater prevalence rate of dysmenorrhea in young women (aged 17–24 years old) and relative late-onset of fibromyalgia (increased prevalence rate after 30 years old) [1,10], cyclic recurrent menstrual pain in dysmenorrhea women may raise the risk of fibromyalgia via the aforementioned mechanisms. Moreover, the altered brain function and structures associated with repetitive pain stimuli may be reversed after pain stimuli cease [22,23,24]. Thus, treatment of dysmenorrhea might reduce the risk of fibromyalgia in dysmenorrhea patients. In the present study, we used the Longitudinal Health Insurance Database (LHID) 2000 from the Taiwan National Health Research Institutes Database (NHIRD) to investigate whether (1) women with dysmenorrhea have higher risk of fibromyalgia and (2) treatment of dysmenorrhea reduced the risk of fibromyalgia.

## 2. Materials and Methods

### 2.1. Data Source

The LHID 2000 consisted of 1 million people who were randomly selected from the NHIRD (covered 99% of the population in Taiwan) in 2000. This nationwide database contained information on medical facility registries, medication prescriptions, and outpatient, inpatient, and emergency visiting data for Taiwan’s general public. For the patients’ privacy, their diagnoses in the claims data of the NHIRD are used for administrative purposes, and anonymity of the data is ensured by assigning identification numbers.

### 2.2. Sample Participant

All protocols were approved by the Institutional Review Board of China Medical University Hospital, Taiwan (CMUH 104-REC2-115). This cohort study was based on the cumulative outpatients and inpatients population from LHID 2000. The inclusion criteria were patients newly diagnosed with dysmenorrhea (ICD-9-CM 625.3) who had two outpatient visits or an inpatient visit, and the study population was followed up from 1 January 2000, to 13 December 2011. We also selected a comparable control cohort of a normal population (without dysmenorrhea). The dysmenorrhea cohort was matched with the comparison cohort at a 1:1 ratio based on gender, age, and the year of entry study by frequency matching. The exclusion criteria were the patients who were aged < 12 years old or who were diagnosed with fibromyalgia (ICD-9-CM 729.1) before entry to the study. The date of diagnosis of dysmenorrhea was used as the index date, and patients were followed up until the appearance of fibromyalgia, death, withdrawal from the insurance program, or the end of the study period (31 December 2011).

### 2.3. Outcome, Comorbidity and Medication

The main end point of the study was fibromyalgia (ICD-9-CM 729.1). The comorbidities analyzed in this study were tobacco use disorder (ICD-9-CM 305.1), obesity (ICD-9-CM 278), menstrual migraine (ICD-9-CM 346.4), depressive disorder (ICD-9-CM 269.2, 269.3 and 311), anxiety disorder (ICD-9-CM 300), and sleep disorder (ICD-9-CM 307.4 and 780.5). The common comorbidities identified were diagnosed based on ICD-9 codes in the LHID 2000 prior to the index date. The influence of medications was also analyzed by recognition of anatomical therapeutic chemical codes in the LHID 2000. We considered the effect of drugs for the risk of the dysmenorrhea patient and listed the drugs as variables, including NSAID (e.g., ibuprofen, ketoprofen, mefenamic acid, and diclofenac), antidepressants (e.g., duloxetine and milnacipran), anticonvulsants (gabapentin and pregabalin), rofecoxib, and hormonal contraceptives (e.g., estrogen and ethinyl estradiol) [7].

### 2.4. Statistical Analysis

The dysmenorrhea cohort was compared with the comparison cohort concerning baseline characteristics. The Chi-squared test was used to compare categorical variables, whereas the 2-sample *t*-test was used to compare continuous variables between the dysmenorrhea cohort and comparison cohort where necessary. We assessed the overall and age-specific incidences of fibromyalgia for the groups with and without dysmenorrhea. Univariable and multivariable Cox proportional hazard regression models were used to assess the risk of fibromyalgia, and the hazard ratio (HR) and the 95% confidence interval (CI) were estimated. A multivariable model was performed by controlling for potential confounding variables, such as age, comorbidities of menstrual migraine, anxiety disorder, sleep disorder, NSAID use, antidepressants use, and hormonal contraceptives use. The differences in the risk of fibromyalgia between the two cohorts was estimated using Kaplan–Meier methods with the log-rank. Analyses were performed using SAS software (version 9.4 for Windows; SAS Institute, Cary, NC, USA) for Windows 10. All statistical significances were set at a *p* < 0.05.

## 3. Results

There were 38,243 subjects in the group with dysmenorrhea and 38,243 subjects in the group without dysmenorrhea, which were identified from the LHID 2000 in this study. Among the dysmenorrhea cohort, 1,991 subjects had endometriosis (ICD-9-CM 617.9). Both of the cohorts had similar proportions of age stratification. The mean ± SD ages of the study subjects were 27.2 ± 9.45 years old in the dysmenorrhea cohort and 27.2 ± 9.32 years old in the comparison cohort (*p* < 0.001). Patients with dysmenorrhea tended to be more likely to have obesity, menstrual migraines, depressive disorders, anxiety disorders and sleep disorders (all *p* < 0.001), relative to the comparison cohort. Patients with dysmenorrhea also had higher proportion of taking NSAID, antidepressants, hormonal contraceptives (all *p* < 0.001) and rofecoxib (*p* < 0.01), relative to the comparison cohort (Table 1). The mean follow-up duration was 7.76 ± 3.65 years in the dysmenorrhea cohort and 7.73 ± 3.65 years in the comparison cohort.

The overall prevalence of fibromyalgia was higher among cases in the dysmenorrhea cohort (939 fibromyalgia cases, 2.45%) than in the comparison cohort (646 fibromyalgia cases, 1.68%). The incidence rate of fibromyalgia was higher in the dysmenorrhea cohort than in the comparison cohort (3.16 vs. 2.19 per 1000 person-years). With adjustment for confounding factors, the HR for the dysmenorrhea cohort was 1.40 (95% CI = 1.26–1.55) compared to the comparison cohort. Compared to the ≤49 years old age group, the group with subjects 50–64 years old and more than 65 years old had a significant (1.47 (95% CI = 1.29–1.67), 2.49 (95% CI = 2.21–2.89), respectively) risk for fibromyalgia. Among all of the comorbidities and medications, patients with menstrual migraine and NSAID use tended to have a higher risk for the development of fibromyalgia (all *p* < 0.05) (Table 2).

**Figure 1:**
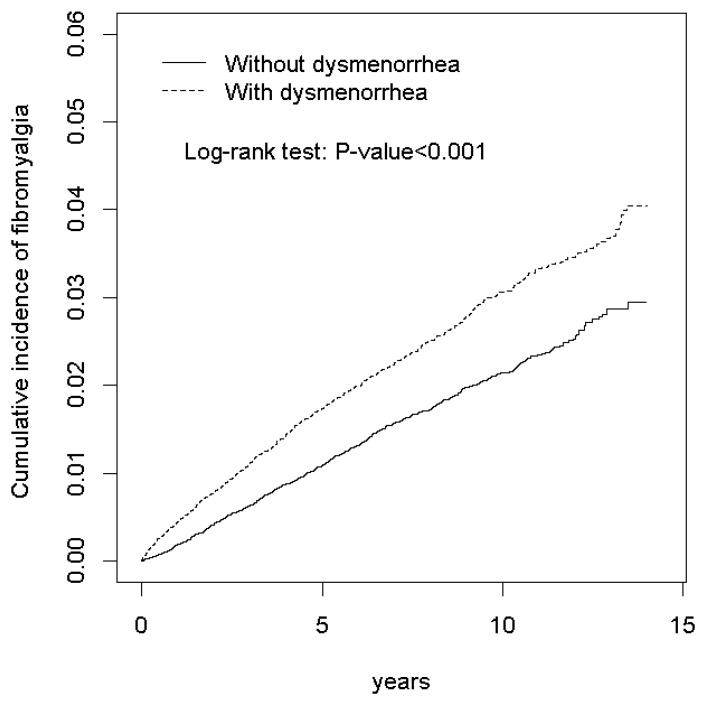
Cumulative incidence comparison of fibromyalgia for patients with (dashed line) or without (solid line) dysmenorrhea.

The Kaplan–Meier analysis revealed that the dysmenorrhea cohort had a higher cumulative incidence of fibromyalgia than the comparison cohort at the end of follow-up (Figure 1; *p* < 0.001). More specifically, after controlling for potential confounding factors, the dysmenorrhea cohort had a significantly higher risk in age and comorbidities stratification than the non-dysmenorrhea cohort. Compared to the non-dysmenorrhea cohort, the risk of fibromyalgia for dysmenorrhea patients who were taking NSAID (adjusted HR = 1.41, 95% CI = 1.21–1.57), not taking antidepressants (adjusted HR = 1.39, 95% CI = 1.25–1.55), taking antidepressants (adjusted = 1.53, 95% CI = 1.10–2.14), not taking hormonal contraceptives (adjusted HR = 1.50, 95% CI = 1.33–1.69) and taking hormonal contraceptives (adjusted HR = 1.22, 95% CI = 1.01–1.46) were significantly higher (Table 3).

Among the dysmenorrhea cohort, 22,195 (58.0%), 28 (0.07%), and 131 (0.3%) subjects were only prescribed with NSAID, antidepressants, and hormonal contraceptives, respectively. There were 10,871 (28.4%), 1991 (5.2%), and 6 (0.01%) subjects prescribed with NSAID plus hormonal contraceptives, NSAID plus antidepressants, and antidepressants plus hormonal contraceptives, respectively. Two thousand and ninety-five (5.47%) subjects were prescribed with all three kinds of medication, and 926 (2.4%) subjects were not prescribed any of the three kinds of medication. For the effect of medications on the risk of fibromyalgia with or without dysmenorrhea, both cohorts (with or without dysmenorrhea) had a significantly higher risk of fibromyalgia as long as they were taking NSAID (adjusted HR = 2.05, 95% CI = 1.54–2.73 and adjusted HR = 1.45, 95% CI = 1.09–1.93). Compared with the patients without dysmenorrhea and not taking antidepressants, the dysmenorrhea patients who were taking antidepressants (adjusted HR = 1.38, 95% CI = 1.11–1.71) and who were not taking antidepressants (adjusted HR = 1.39, 95% CI = 1.25–1.55) also had a significant higher risk of fibromyalgia. Similarly, compared with the patients without dysmenorrhea and not taking hormonal contraceptives, the dysmenorrhea patients who were taking hormonal contraceptives (adjusted HR = 1.31, 95% CI = 1.13–1.52) and who were not taking hormonal contraceptives (adjusted HR = 1.49, 95% CI = 1.32–1.69) had a significantly higher risk of fibromyalgia. However, dysmenorrhea patients who were taking hormonal contraceptives had a significantly lower risk of fibromyalgia than who were not taking hormonal contraceptives (Table 4).

## 4. Discussion

In the present study, we conducted a cohort study using LHID 2000 to investigate whether treatment of dysmenorrhea can reduce the risk of fibromyalgia or not. After controlling confounding variables, our results revealed that women with dysmenorrhea have a significantly higher risk of fibromyalgia than women without dysmenorrhea. Although treatment of dysmenorrhea with NSAID or antidepressants have not significantly reduced the risk of fibromyalgia on women with dysmenorrhea, treatment with hormonal contraceptives has significantly reduced the risk on fibromyalgia on women with dysmenorrhea. These results indicated that dysmenorrhea may be a risk factor of fibromyalgia, whereas treatment of dysmenorrhea with hormonal contraceptives may reduce the risk of fibromyalgia.

As we hypothesized, women with dysmenorrhea were revealed to be at a higher risk of fibromyalgia than women without dysmenorrhea. Despite the elder age, having menstrual migraine, and taking NSAID was also associated with a higher risk of fibromyalgia; the dysmenorrhea cohort still had a 40% higher risk of fibromyalgia than the non-dysmenorrhea cohort after controlling confounding variables. The result from Kaplan–Meier analysis further revealed that the dysmenorrhea cohort had a higher incidence of fibromyalgia than the non-dysmenorrhea cohort across the follow-up period. These results indicated that dysmenorrhea may be a risk factor of fibromyalgia. Previous studies reported that women with fibromyalgia had higher prevalence rate of dysmenorrhea than the women with arthritis and the healthy controls [16,17]. A community-based telephone survey study with a larger sample size also reported that women with fibromyalgia have a higher prevalence rate of dysmenorrhea than women without fibromyalgia [25]. The tender point number on women with fibromyalgia was significantly higher in patients with dysmenorrhea than patients without dysmenorrhea [26]. Thus, the cyclic recurrent menstrual pain in dysmenorrhea might be a risk factor of fibromyalgia, which may contribute to the higher prevalence rate of fibromyalgia in women than in men.

However, only treatment with hormonal contraceptives on dysmenorrhea can significantly reduce the risk of fibromyalgia. Neither treatment with NSAID or treatment with antidepressants on dysmenorrhea can reduce the risk of dysmenorrhea. These results may partly be due to a bias; the records of dysmenorrhea patients in the LHID 2000 may represent a population with moderate to severe dysmenorrhea. Previous studies have reported that the majority of women with dysmenorrhea do not seek health care from physicians and tend to self-manage the condition [27,28,29]. For the patients who did seek health care from physicians, the symptoms of dysmenorrhea were more severe than the patients who did not seek health care from physicians [28,30,31]. The severity of dysmenorrhea may influence the treatment strategy from the physicians. The first-line pharmacological treatment for dysmenorrhea is NSAID. If the NSAID treatment is ineffective to manage dysmenorrhea, treatment with hormonal contraceptives or antidepressants can be considered [6,7]. One study has reported that both treatment with NSAID and with oral contraceptive pills has less effectiveness for treating severe dysmenorrhea than mild dysmenorrhea [8]. Thus, the possible population bias of dysmenorrhea in the LHID 2000 may explain the higher risk of fibromyalgia in the dysmenorrhea cohort than the non-dysmenorrhea cohort, even with the treatment of NSAID, antidepressants or hormonal contraceptives. On the other hand, the risk of fibromyalgia was significantly decreased when dysmenorrhea was treated with hormonal contraceptives compared to treatment without hormonal contraceptives. Considering that the treatment of hormonal contraceptives is the second-line treatment of primary dysmenorrhea and first-line treatment of secondary dysmenorrhea [6], the treatment with hormonal contraceptives may be effectively treatment in primary dysmenorrhea patient who fail to respond to the treatment of NSAID and in secondary dysmenorrhea patients who have endometriosis, which then decreased the risk of fibromyalgia.

Several limitations in the present study should be noted. Firstly, the LHID 2000 lacks detailed patient information (e.g., lifestyles, nutrition status, and physical, mental and laboratory examinations) which may also influence the risk of fibromyalgia, although the strangest risk factors have been partially controlled in the present study. Secondly, we used ICD-9-CM code to identify the dysmenorrhea and fibromyalgia, without any information about the severity of dysmenorrhea and fibromyalgia. The confounding by indication (treatment procedural considered by the baseline severity) may bias the medication prescription, although most dysmenorrhea patients have been prescribed with NSAID and NSAID plus hormonal contraceptives. Thirdly, no information for the treatment efficacy of dysmenorrhea was available in the LHID 2000, which prevented further evaluation for the effect of medication on the risk of fibromyalgia in dysmenorrhea patients. Future prospective studies should be conducted to identify the causes and prevention strategies in detail.

## 5. Conclusions

In conclusion, our results demonstrated that women with dysmenorrhea have a higher risk of fibromyalgia than women without dysmenorrhea, indicating that dysmenorrhea may be a risk factor of fibromyalgia. However, only treatment of dysmenorrhea with hormonal contraceptives reduced the risk of fibromyalgia, suggested that personalized medicine for treatment of dysmenorrhea may be important to reduce the risk of fibromyalgia. Future studies are needed to identify the causes and prevention strategies in detail.

## Figures and Tables

**Table 1:** Demographic characteristics and comorbidities in cohorts with and without dysmenorrhea.

	Dysmenorrhea		*p*-Value
	No (N = 38,243)	Yes (N = 38,243)	
Age			0.99
≤49	18,423 (48.2%)	18,428 (48.2%)	
50–64	11,087 (29.0%)	11,087 (29.0%)	
65+	8728 (22.8%)	8728 (22.8%)	
Mean ± SD ^†^	27.2 ± 9.45	27.2 ± 9.32	<0.001
Comorbidity			
Tobacco use disorder	96 (0.25%)	117 (0.31%)	0.15
Obesity	387 (1.01%)	588 (1.54%)	<0.001
Menstrual migraine	590 (1.54%)	1318 (3.45%)	<0.001
Depressive disorder	818 (2.14%)	1184 (3.10%)	<0.001
Anxiety disorder	2164 (5.66%)	3572 (9.34%)	<0.001
Sleep disorder	2802 (7.33%)	5662 (14.8%)	<0.001
Medication			
NSAID	34,639 (90.6%)	37,152 (97.2%)	<0.001
Antidepressants	2862 (7.48%)	4120 (10.8%)	<0.001
Anticonvulsants	50 (0.13%)	45 (0.12%)	0.61
Hormonal contraceptives	9092 (23.8%)	13,103 (34.3%)	<0.001
Rofecoxib	54 (0.14%)	84 (0.22%)	0.01

All categorical variables were tested by Chi-Squared Test. ^†^: tested by 2-sample *t*-test; SD: standard deviation; NSAID: nonsteroidal anti-inflammatory drugs.

**Table 2:** Incidence and hazard ratio for the risk factors of fibromyalgia.

Variable	Event	PY	Rate	Crude HR (95% CI)	Adjusted HR (95% CI)
Dysmenorrhea					
No	646	295,539	2.19	1.00	1.00
Yes	939	296,925	3.16	1.45 (1.31, 1.60) ***	1.40 (1.26, 1.55) ***
Age, year					
≤49	530	295,006	1.80	1.00	1.00
50–64	452	167,646	2.70	1.50 (1.32, 1.70) ***	1.47 (1.29, 1.67) ***
65+	603	129,812	4.65	2.57 (2.29, 2.89) ***	2.4 (2.21, 2.82) ***
Comorbidity					
Tobacco use disorder					
No	1582	591,493	2.67	1.00	1.00
Yes	3	971	3.09	1.10 (0.36, 3.41)	NE
Obesity					
No	1564	586,142	2.67	1.00	1.00
Yes	21	6322	3.32	1.23 (0.80, 1.89)	NE
Menstrual migraine					
No	1520	579,465	2.62	1.00	1.00
Yes	65	12,999	5.00	1.89 (1.47, 2.42) ***	1.39 (1.08, 1.80) *
Depressive disorder					
No	1540	579,230	2.66	1.00	1.00
Yes	45	13,234	3.40	1.26 (0.94, 1.70)	NE
Anxiety disorder					
No	1418	551,049	2.57	1.00	1.00
Yes	167	41,415	4.03	1.56 (1.33, 1.83) ***	1.10 (0.92, 1.33)
Sleep disorder					
No	1364	536,965	2.54	1.00	1.00
Yes	221	55,499	3.98	1.54 (1.34, 1.78) ***	1.07 (0.92, 1.25)
Medication					
NSAID					
No	68	45,460	1.50	1.00	1.00
Yes	1517	547,004	2.77	1.82 (1.43, 2.32) ***	1.55 (1.21, 1.99) ***
Antidepressants					
No	1418	544,904	2.60	1.00	1.00
Yes	167	47,560	3.51	1.33 (1.14, 1.57) ***	0.97 (0.81, 1.16)
Anticonvulsants					
No	1585	592,036	2.68	1.00	1.00
Yes	0	428	0.00	NE	NE
Hormonal contraceptives					
No	1078	429,309	2.51	1.00	1.00
Yes	507	163,155	3.11	1.23 (1.11, 1.37) ***	0.97 (0.87, 1.09)
Rofecoxib					
No	1584	591,491	2.68	1.00	1.00
Yes	1	973	1.03	0.38 (0.05, 2.69)	NE

PY: total person-year; Rate, incidence rate, per 1000 person-years; Crude HR, relative hazard ratio; Adjusted HR: hazard ratio from multivariable analysis including age, comorbidities of menstrual migraine, anxiety disorder, sleep disorder, and medication of NSAID, antidepressants, and hormonal contraceptives as confounding variables. CI: confidence interval; NSAID: nonsteroidal anti-inflammatory drugs; NE: not estimable; *: *p* < 0.05; ***: *p* < 0.001.

**Table 3:** Incidence and hazard ratio of fibromyalgia for patients with or without dysmenorrhea.

	Dysmenorrhea						Dysmenorrhea to Non-Dysmenorrhea	
Variables	No			Yes				
	Event	PY	Rate	Event	PY	Rate	Crude HR (95% CI)	Adjusted HR (95% CI)
Stratify age								
≤49	213	147,126	1.45	317	147,880	2.14	1.48 (1.25, 1.76) ***	1.41 (1.18, 1.68) ***
50–64	175	83,458	2.10	277	84,189	3.29	1.57 (1.30, 1.90) ***	1.50 (1.23, 1.82) ***
65+	258	64,956	3.97	345	64,856	5.32	1.34 (1.14, 1.57) ***	1.30 (1.10, 1.53) **
Comorbidity ^‡^								
No	552	265,624	2.08	702	239,716	2.93	1.41 (1.26, 1.58) ***	1.42 (1.27, 1.59) ***
Yes	94	29,915	3.14	237	57,208	4.14	1.33 (1.05, 1.69) *	1.37 (1.07, 1.74) *
Medication								
NSAID								
No	51	34,567	1.48	17	10,894	1.56	1.06 (0.61, 1.83)	1.14 (0.65, 2.00)
Yes	595	260,973	2.28	922	286,031	3.22	1.42 (1.28, 1.57) ***	1.41 (1.27, 1.57) ***
Antidepressants								
No	595	276,548	2.15	823	268,356	3.07	1.43 (1.28, 1.58) ***	1.39 (1.25, 1.55) ***
Yes	51	18,991	2.69	116	28,568	4.06	1.53 (1.10, 2.13) *	1.53 (1.10, 2.14) *
Hormonal contraceptives								
No	467	230,959	2.02	611	198,351	3.08	1.52 (1.35, 1.72) ***	1.50 (1.33, 1.69) ***
Yes	179	64,581	2.77	328	98,573	3.33	1.22 (1.01, 1.46) *	1.22 (1.01, 1.46) *

PY: total person-year; Rate, incidence rate, per 1000 person-years; Crude HR, relative hazard ratio; Adjusted HR: hazard ratio from multivariable analysis including age, comorbidities of menstrual migraine, anxiety disorder, sleep disorder, and medication of NSAID, antidepressants, and hormonal contraceptives as confounding variables. CI: confidence interval; NSAID: nonsteroidal anti-inflammatory drugs; ^‡^: subjects with any one of the comorbidities were classified as the comorbidity group; *: *p* < 0.05; **: *p* < 0.01; ***: *p* < 0.001.

**Table 4:** The effect of medication on the risk of fibromyalgia with or without dysmenorrhea.

Variables		Event	PY	Rate	Adjusted HR (95% CI)	*p* for Interaction
Dysmenorrhea	NSAID					0.31
No	No	51	34,567	1.48	1 (Reference)	
No	Yes	595	260,973	2.28	1.45 (1.09, 1.93) *	
Yes	No	17	10,894	1.56	1.11 (0.64, 1.93)	
Yes	Yes	922	286,031	3.22	2.05 (1.54, 2.73) ***	
Dysmenorrhea	Antidepressant					0.72
No	No	595	276,548	2.15	1 (Reference)	
No	Yes	51	18,991	2.69	0.91 (0.68, 1.22)	
Yes	No	823	268,356	3.07	1.39 (1.25, 1.55) ***	
Yes	Yes	116	28,568	4.06	1.38 (1.11, 1.71) ***	
Dysmenorrhea	Hormonal contraceptives					0.04
No	No	467	230,959	2.02	1 (Reference)	
No	Yes	179	64,581	2.77	1.09 (0.92, 1.30)	
Yes	No	611	198,351	3.08	1.49 (1.32, 1.69) ***	
Yes	Yes	328	98,574	3.33	1.31 (1.13, 1.52) ***	

PY: total person-year; Rate: incidence rate, per 1000 person-years; Adjusted HR: hazard ratio from multivariable analysis including age, comorbidities of menstrual migraine, anxiety disorder, sleep disorder, and medication of NSAID, antidepressants, and hormonal contraceptives as confounding variables. CI: confidence interval; NSAID: nonsteroidal anti-inflammatory drugs; *: *p* < 0.05; ***: *p* < 0.001.
